# Hydrous Molybdenum Oxide Coating of Zinc Metal Anode via the Facile Electrodeposition Strategy and Its Performance Improvement Mechanisms for Aqueous Zinc−Ion Batteries

**DOI:** 10.3390/molecules29133229

**Published:** 2024-07-08

**Authors:** Jianwei Yuan, Yutao Shi, Weibai Bian, Huaren Wu, Yingjun Chen, Chengcheng Zhou, Xiaohui Chen, Wei Zhang, Hailin Shen

**Affiliations:** School of Chemical Engineering and Materials, Changzhou Institute of Technology, Changzhou 213022, China; yuanjw@czust.edu.cn (J.Y.);

**Keywords:** electrodeposition, anode, HMoO_x_ coating, protection, aqueous zinc−ion battery

## Abstract

Aqueous zinc−ion batteries (ZIBs) are widely recognized as highly promising energy storage devices because of their inherent characteristics, including superior safety, affordability, eco−friendliness, and various other benefits. However, the significant corrosion of the zinc metal anode, side reactions occurring between the anode and electrolyte, and the formation of zinc dendrites significantly hinder the practical utilization of ZIBs. Herein, we utilized an electrodeposition method to apply a unique hydrous molybdenum oxide (HMoO_x_) layer onto the surface of the zinc metal anode, aiming to mitigate its corrosion and side reactions during the process of zinc deposition and stripping. In addition, the HMoO_x_ layer not only improved the hydrophilicity of the zinc anode, but also adjusted the migration of Zn^2+^, thus facilitating the uniform deposition of Zn^2+^ to reduce dendrite formation. A symmetrical cell with the HMoO_x_−Zn anode displayed reduced−voltage hysteresis (80 mV at 2.5 mA/cm^2^) and outstanding cycle stability after 3000 cycles, surpassing the performance of the uncoated Zn anode. Moreover, the HMoO_x_−Zn anode coupled with a γ−MnO_2_ cathode created a considerably more stable rechargeable full battery compared to the bare Zn anode. The HMoO_x_−Zn||γ−MnO_2_ full cell also displayed excellent cycling stability with a charge/discharge−specific capacity of 129/133 mAh g^−1^ after 300 cycles. In summary, this research offers a straightforward and advantageous approach that can significantly contribute to the future advancements in rechargeable ZIBs.

## 1. Introduction

As coal and other fossil fuel resources have been excessively depleted, a plethora of new rechargeable batteries have emerged, including lithium−ion batteries (LIBs) [1], zinc−ion batteries (ZIBs) [2], magnesium−ion batteries [3], sodium−ion batteries [4], and aluminum−ion batteries [5,6,7], revolutionizing the way we power portable electronic devices and vehicles. Among the emerging battery technologies, aqueous ZIBs are particularly favored for the extensive energy storage applications owing to their notable features, including enhanced safety, affordability, lack of pollution, convenient assembly, and high specific/volumetric capacity [8,9,10,11,12].

Conventional ZIBs are assembled by the cathode (MnO_2_) and anode (Zn metal), which show poor cycling stability. The performance of ZIBs heavily relies on the zinc anode, which faces numerous challenges. Side reactions, such as corrosion and the hydrogen evolution reaction (HER) during the charging and discharging processes, are a key factor affecting the performance of the Zn anode. Zn is prone to corrosion when exposed to the electrolyte, resulting in the formation of zinc oxide or hydroxide, and HER leads to the loss of active material and decreased battery efficiency [13,14,15]. More importantly, the uncontrolled growth of dendrites can occur during the charging/discharging processes, which are needle−like structures that can penetrate the separator and result in short circuits, thus posing safety risks. Hence, for the successful commercialization of ZIBs, it is imperative to explore effective strategies to protect the zinc anode by controlling the side reactions and dendrite growth. To date, numerous effective strategies have been suggested to tackle the aforementioned concerns, including zinc alloying [16], three−dimensional structure design [17,18], surface modification [19,20], electrolyte modification [21,22,23], separator design [24], etc. Among them, surface coating is considered a simple and effective strategy. The coating materials for modifying the Zn anode include carbons (e.g., graphene oxide [25], carbon black [26], and carbon nanotubes [27]), metal compounds (e.g., ZnS [28], Al_2_O_3_ [29], CaCO_3_ [19], and ZrO_2_ [30]), organic polymers (e.g., β−phase poly(vinylidene difluoride) (β−PVDF) [31], polyamide ((PA)@Zn(TfO)_2_) [32], polypyrrole (PPy) [33]), MOFs (zeolitic imidazolate framework−8 (ZIF−8) [34], zeolitic imidazolate framework−7 (ZIF−7) [35], and Universistetet/Oslo−66 (UIO−66) MOFs [36]). The inorganic coatings are more effective at restraining dendrite growth because of their physical characteristics, such as high mechanical strength and rigidity, whereas the polymer coating is susceptible to puncture by dendrites due to its flexibility [37].

Previously, Liu et al. proposed an amorphous MoO_x_ electrode as the anode material for aqueous NH_4_^+^−ion batteries, which displayed high gravimetric/areal capacities of 175 mAh g^−1^ at 0.22 A g^−1^. The spectroscopic investigations carried out to understand the charge storage mechanism of MoO_x_ indicate that the MnO_x_ anode facilitates the rapid transportation of NH_4_^+^ via hydrogen bond formation and breaking [38]. In addition, a few examples (e.g., MoO_2_/carbon [39] and MoO_3−x_/Mxene [40]) have been recently employed to investigate Zn^2+^ storage behavior because of their inherent multiple valence states, along with significant theoretical capacity, electron−conducting ability, and robust structural stability. Molybdenum oxides, as a stable oxide and electrode material, can provide a more stable interface, but can also improve electron and ion transport, and improve reaction kinetics; hence, molybdenum oxides can be used as a protective layer of a Zn negative electrode. The electrochemical properties of the Zn anode modified by a molybdenum oxide layer are far from what were expected. In contrast to the hydrophobic layer, the hydrophilic layer can be selective when modifying the transport characteristics of Zn^2+^, thus improving the transport efficiency of zinc ions. The hydrophilic surface can also improve the contact between the electrolyte and the electrode, helping Zn^2+^ to deposit more evenly on the anode’s surface. In addition, the hydrophilic layer helps to reduce electrolyte decomposition, hydrogen release, and other side reactions. For example, Li et al. discovered that a TiO_2_−coated Zn anode exhibited improved surface wettability compared to the uncoated Zn. This enhancement facilitated the movement and even the deposition of Zn^2+^, while also reducing the charge transfer resistance [41]. Therefore, the HMoO_x_ layer combines the advantages of molybdenum oxide and the hydrophilic surface, which can effectively inhibit the corrosion, side reactions, and zinc dendrite growth during zinc deposition/stripping. To our knowledge, there are no relevant studies on HMoO_x_ as a protective coating applied onto the surface of zinc at present.

There are various methods to establish a protective layer on the zinc anode’s surface, primarily focusing on physical coating and in situ synthesis. Unlike them, the electrodeposition method is a relatively simple and easy−to−operate technique. The thickness, uniformity, and consistency of a protective film can be precisely controlled by adjusting the deposition parameters, which can enhance the performance consistency of the battery. Noori et al. reported that the electrodeposition method can produce the thinnest and most uniform few−layer, thin MoS_2_ films on a graphene electrode [42]. Bhoyate et al. employed an electrochemical deposition method to produce a distinctive 2D MoS_2_ layer on a zinc anode [43]. Cao et al. have successfully electrochemically deposited MoO_2_ from an ammonium molybdate solution and systematically explored how variations in electrolyte composition and deposition conditions impact the characteristics of MoO_2_ [44].

Herein, we present a rapid and straightforward method for coating the surface of the Zn anode with HMoO_x_ via constant potential deposition. By optimizing the deposition time, the final oxidation layer with the best performance is obtained, and the film thickness can be finely adjusted on the nanometer scale. This demonstrates a significant improvement compared with the bare Zn, and the results show that based on the metal surface forming a thin zinc molybdenum oxide hydrate layer, there is an even distribution of the lead zinc metal surface electric field. The HMoO_x_ coating aids in enhancing the migration of Zn^2+^ ions, ensuring uniform deposition, and consequently enhancing the overall performance of the battery. The HMoO_x_−coated Zn foil (denoted as HMoO_x_−Zn) was utilized to assemble the symmetric cells, which can display a more stable cycle stability and reduced−voltage hysteresis of ~103 mV than the bare Zn anode at 2.5 mA cm^−2^. Simultaneously, the HMoO_x_−Zn||γ−MnO_2_ full cell also exhibited exceptional stability over numerous cycles, maintaining a charge/discharge−specific capacity of 129/133.1 mAh g^−1^ after 300 cycles. This work, therefore, offers valuable insights into the development of a highly reversible Zn anode.

## 2. Results and Discussion

**Figure 1:**
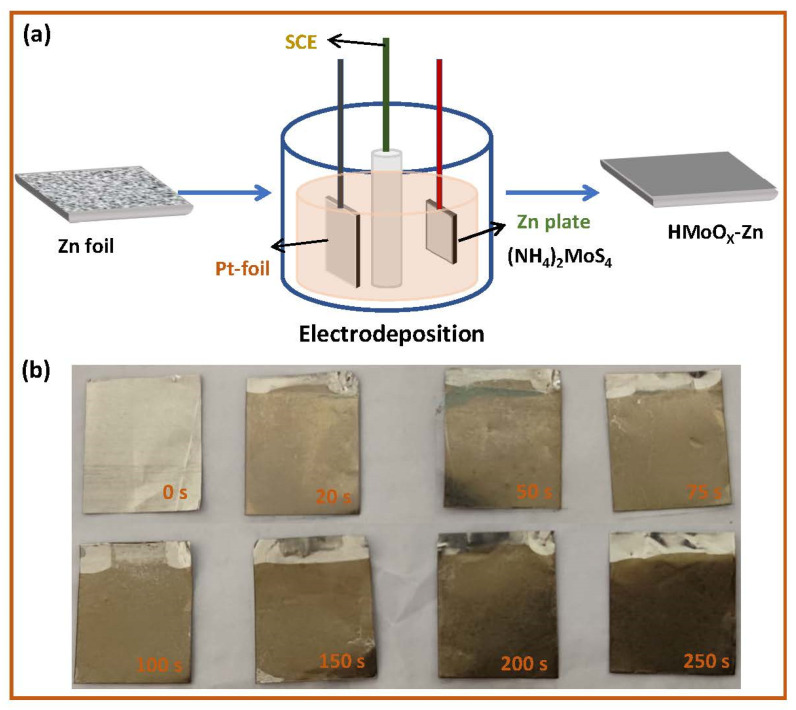
The preparation of the HMoO_x_−Zn anode. (**a**) The synthesis process of the HMoO_x_−Zn anode via electrodeposition. (**b**) The digital pictures of the HMoO_x_−Zn anode at different deposition times (0 s, 20 s, 50 s, 75 s, 100 s, 150 s, 200 s, and 250 s).

The HMoO_x_−Zn anode was prepared throughout this project using a simple constant−potential electro−deposition method, as shown in Figure 1a. At −0.8 V vs. SCE, the (NH_4_)_2_MoS_4_ solution with a concentration of 5 mM began to undergo reduction on the surface of Zn, forming MoO_4_^2−^ ions [44]. As shown in Figure 1b, the deposition time of the reduction process was altered to adjust the thickness of the HMoO_x_ layer, and the HMoO_x_−Zn anode surface presented a distinct color compared to the bare zinc. With the increase in the deposition time, the surface color of the HMoO_x_−Zn anode deepened, which is consistent with the theoretical inference that the HMoO_x_ coating is thicker with a longer deposition time.

**Figure 2:**
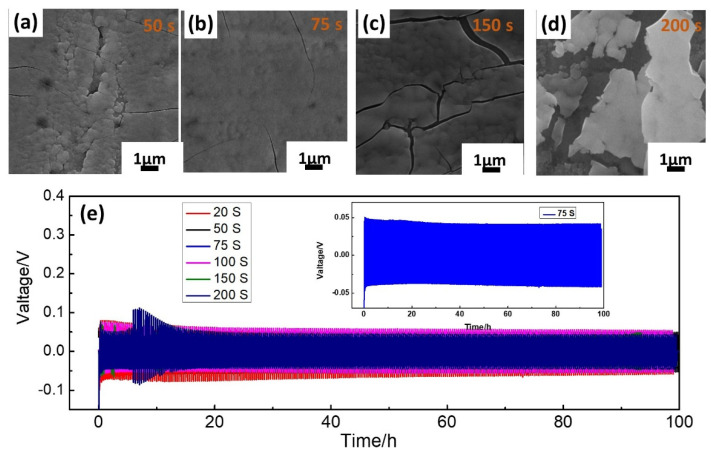
The optimization of preparation of the HMoO_x_−Zn anode. (**a**–**d**) The SEM of the HMoO_x_−Zn anode at different deposition times of 50 s, 75 s, 150 s, and 250 s, respectively. (**e**) The dynamic measurements of a symmetric cell for HMoO_x_−Zn anodes for 20 s, 50 s, 75 s, 100 s, 150 s, and 200 s in stripping/plating cycles (300 cycles, 2.5 mA cm^−2^), respectively. The illustration in Figure 2e shows magnified information corresponding to 75 s. The stripping/plating time of anodes was 10 min for each cycle.

The effect of electrodeposition varied with changes in the duration of the reduction process’s deposition, as illustrated in Figure 2a–d and Figure S1. The SEM images illustrated that the increase in the electrodeposition time resulted in an increase in the incidence of cracking in the HMoO_x_ layer. The electrodeposition time of 75 s was considered as an optimum time for a uniform HMoO_x_ coating on the Zn surface, while at 250 s, the HMoO_x_ layer demonstrated non−uniform fragments and higher surface resistance. Galvanostatic charge/discharge measurements were conducted on symmetrical batteries to evaluate the effect of the ammonium molybdate electrodeposition time on the electrochemical properties of HMoO_x_−Zn anodes in Figure 2e and Figure S2. The HMoO_x_−Zn anode showed excellent cycle stability when the deposition time reached 20 s; however, its stripping/deposition voltage range was wide. With the increase in the electrochemical deposition time, the charge–discharge voltage range narrowed until the deposition time was 75 s, and the voltage difference was at its narrowest. However, the stability of the HMoO_x_−Zn anode diminished with the prolonged deposition time. The performance results align with the SEM analysis described earlier. Therefore, the electrodeposition time of 75 s was considered the optimal duration for uniformly applying the HMoO_x_ coating on the Zn surface.

**Figure 3:**
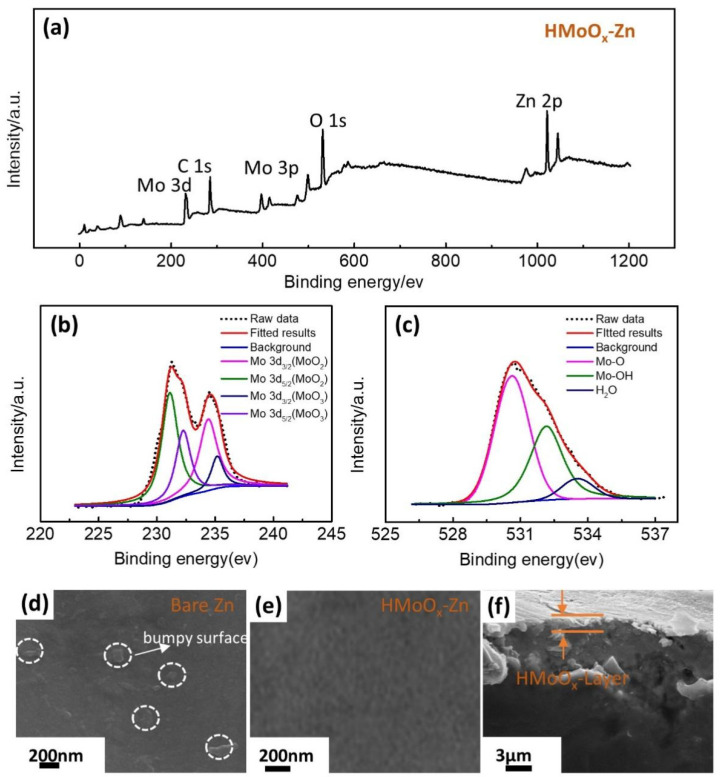
The composition and morphology of analysis for HMoO_x_−Zn. (**a**) The XPS survey spectrum of HMoO_x_−Zn. The high−resolution XPS spectroscopy measurements of the Mo 2p (**b**) and O 1s (**c**). The SEM images of bare Zn (**d**) and HMoO_x_−Zn (**e**). (**f**) The cross−sectional SEM image of the HMoO_x_−Zn.

To analyze the surface elemental composition of HMoOx−Zn, XPS was utilized to measure the sample. As depicted in Figure 3a, the displayed spectrum peaks were attributed to the elements depicted, including Zn, O, Mo, and C. The peaks associated with Zn2p3/2 and Zn2p1/2 were observed at 1021.69 eV and 1046.03 eV, respectively [45]. The splitting of Mo 3d peaks were detected at 232.2 eV for Mo^6+^ 3d_5/2_, 235.1 eV for Mo^6+^ 3d_3/2_, 231.1 eV for Mo^4+^ 3d_5/2_, and 234.4 eV for Mo^4+^ 3d_3/2_ in Figure 3b [46]. Additionally, the O 1s peaks at 530.6 eV, 532.2 eV, and 533.4 eV for HMoO_x_−Zn were fitted, attributed to Mo−O−Mo, Mo−O−H, and H−O−H in Figure 3c, respectively [46,47]. In addition, the top−view SEM images provided insight into the morphological attributes of the bare Zn in Figure 3d and HMoO_x_−Zn in Figure 3e. The bare Zn exhibited an uneven surface with a particulate structure, potentially amplifying dendrite formation, thus further intensifying the decline in cyclic performance. As shown in Figure 3f and Figure S3, there was a uniform and smooth layer of HMoO_x_ on the surface of the HMoO_x_−Zn foil. It was reported that the protective layer can enhance the stability of the anode interface and promote the formation of a stable electrolyte/electrode interface [41].

**Figure 4:**
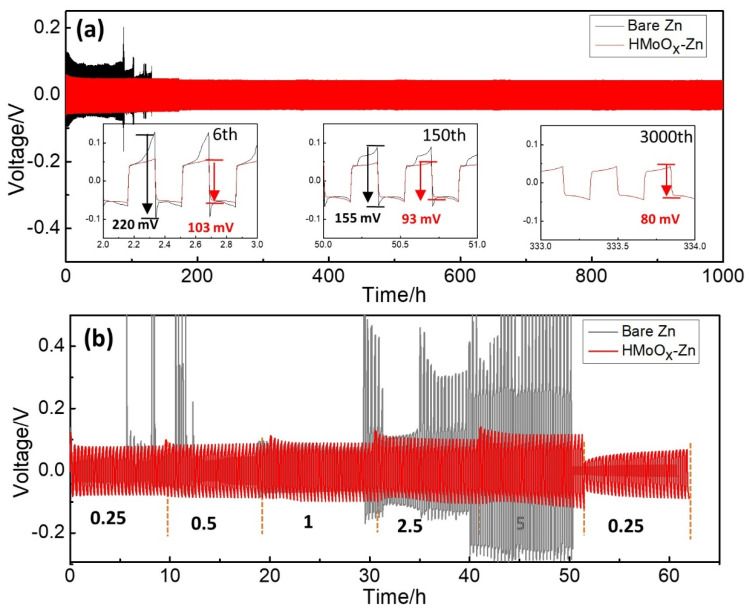
Electrochemical performance of the HMoO_x_−Zn symmetrical cell. (**a**) The dynamic measurements of a symmetric cell for the bare Zn and HMoO_x_−Zn anodes after 3000 cycles (2.5 mA/cm^2^). (**b**) The symmetrical cell test for the bare Zn and HMoO_x_−Zn anodes at various current densities of 0.25, 0.5, 1, 2.5, 5, and 0.25 mA cm^−2^. The stripping/plating time of the anodes was 10 min for each cycle.

The electrochemical plating/stripping characteristics of zinc were examined using symmetric Zn||Zn and HMoO_x_−Zn||HMoO_x_−Zn half cells, as shown in Figure 4a. The bare Zn electrodes (230 mV) showed a significantly higher overpotential than the HMoO_x_−Zn anodes (150 mV). In addition, the bare Zn−based symmetric cells experienced failure after several limited cycles due to noticeable voltage fluctuations and irregular voltage hysteresis. Conversely, the HMoO_x_−coated anodes demonstrated remarkable endurance, maintaining an exceptionally low−voltage hysteresis of 80 mV at 2.5 mA cm^−2^ for over 3000 cycles. The overpotential of the HMoO_x_−Zn anodes (220 mV) was also lower compared to that of the bare Zn (103 mV) at the 16th cycle. This suggested that the introduction of the HMoO_x_ coating could effectively suppress dendrite formation and mitigate side reactions taking place at the interface between the electrode and electrolyte [19,29]. Moreover, conducting continuous galvanostatic charge/discharge over a range of current densities from 0.25 to 5 mA cm^−2^ was employed to assess the rate capability of the two electrodes, as shown in Figure 4b. With increasing current density, the HMoO_x_−Zn anodes exhibited a stable voltage plateau and minimal voltage hysteresis, which could be attributed to the protective HMoO_x_ layer. In contrast, bare Zn electrodes demonstrated an erratic voltage distribution and significant voltage hysteresis. HMoO_x_−Zn anodes possessed a voltage hysteresis of 220 mV without significant voltage fluctuations at an ultra−high current density of 10.0 mA cm^−2^. The exceptional rate capability and reversibility observed in HMoO_x_−Zn anodes could be attributed to the minimized dendrite formation, uniform zinc deposition/stripping, and improved corrosion resistance achieved with the HMoO_x_ coating. Therefore, the aforementioned results indicated that the HMoO_x_ coating could substantially enhance the cycling stability of zinc anodes in aqueous electrolytes.

**Figure 5:**
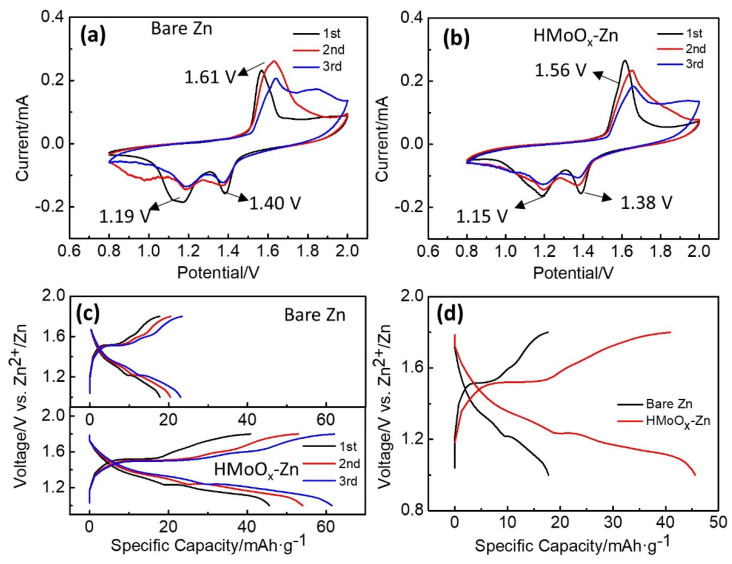
Comparison of electrochemical performance for HMoOx−Zn (or bare Zn)||γ−MnO_2_ full cells using the ZnSO_4_ electrolyte. (**a**,**b**) CV curves for bare Zn||γ−MnO_2_ and HMoO_x_−Zn||γ−MnO_2_ full cells with the voltage window of 0.8–2.0 V at a sweep rate of 0.2 mV s^−1^ in the third cycle, respectively. (**c**) The voltage profiles for bare Zn||γ−MnO_2_ full cells and HMoO_x_−Zn||γ−MnO_2_ between 1.0 and 1.8 V at 0.1 A g^−1^. (**d**) Charge/discharge profiles of the bare Zn||γ−MnO_2_ and HMoO_x_−Zn||γ−MnO_2_ full cells at 0.1 A g^−1^.

Assembled full cells were utilized to explore the effect of the HMoO_x_ coating on the electrochemical behavior within ZIB systems. From observing the cyclic voltammogram (CV) curves in Figure 5a,b, comparing Zn||γ−MnO_2_ to HMoO_x_−Zn||γ−MnO_2_ full cells, the peak positions had shifted slightly. This indicated that the introduction of a HMoOx layer can change the electrochemical properties and kinetic properties of a bare Zn electrode surface, leading to shifts in the oxidation and reduction peaks. Importantly, the Zn||γ−MnO_2_ and HMoO_x_−Zn||γ−MnO_2_ full cells showed similar redox behavior, thus indicating the negligible influence of the HMoO_x_ layer on the redox reaction mechanism in the battery system. Furthermore, HMoO_x_−Zn exhibited a higher peak current density, in comparison to the bare Zn, suggesting that the HMoO_x_ layer could promote electrochemical reactivity and capacity [48]. As exhibited in Figure 5c, the galvanostatic charge–discharge profiles of both batteries were in accordance with the CV curve analysis. In addition, the voltage difference in the HMoO_x_−Zn||γ−MnO_2_ full cell was obviously lower than that in the Zn||γ−MnO_2_ full cell, as shown in Figure 5d. This implies a reduction in the energy required for ion extraction and insertion from the host lattice, which partly mitigated polarization and facilitated electrochemical reactions [49]. Additionally, it underscored the role of the HMoO_x_ coating in regulating and protecting the Zn anode’s surface, thereby boosting the overall performance of the cells.

**Figure 6:**
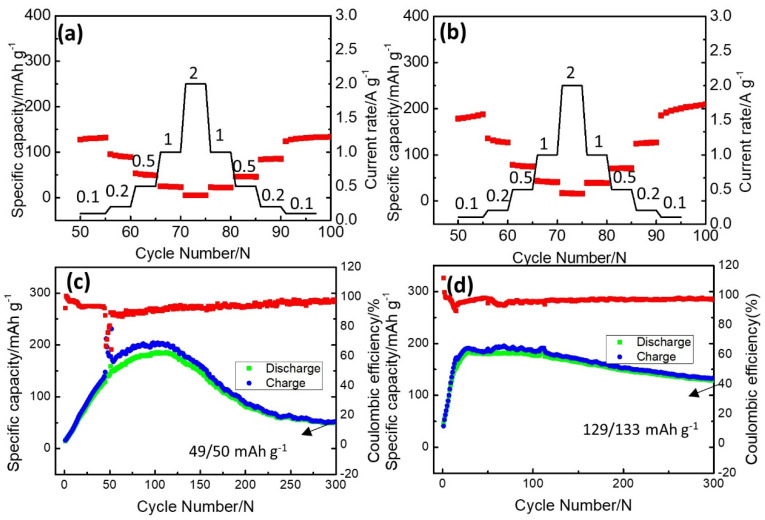
Comparison of cycle performance for HMoO_x_−Zn. (**a**,**b**) Rate performance at 0.1 A g^−1^, 0.2 A g^−1^, 0.5 A g^−1^, 1.0 A g^−1^, and 2.0 A g^−1^ for the bare Zn||γ−MnO_2_ full cell and HMoO_x_−Zn||γ−MnO_2_ full cell, respectively. (**c**,**d**) Cyclic stability for the bare Zn||γ−MnO_2_ full cell and HMoO_x_−Zn||γ−MnO_2_ full cell at 0.1 A g^−1^, respectively (The red curve represents the corresponding coulomb efficiency).

Further validation of the improved cycle performance for the HMoO_x_−Zn||γ−MnO_2_ full cells was achieved using charge–discharge tests, with various current densities ranging from 0.1 to 2.0 A g^−1^. As depicted in Figure 6a,b, the capacity of both samples rapidly diminished as the current densities increased, owing to electrochemical polarization. Nevertheless, it is noteworthy that the rate performance of the HMoO_x_−Zn||γ−MnO_2_ full cell exceeded that of the bare Zn||γ−MnO_2_ full cell across various current densities. The specific capacity of the full cells rebounded when the current density returned to 0.2 A g^−1^, whereas the bare Zn||γ−MnO_2_ full cell exhibited a declining trend in subsequent cycling processes. Simultaneously, the cyclic stability for the bare Zn||γ−MnO_2_ and HMoO_x_−Zn||γ−MnO_2_ full cells was tested at a relatively high current density of 0.1 A g^−1^, as shown in Figure 6c,d. The rapid deterioration in capacity of the bare Zn||γ−MnO_2_ full cell, with only 50 mA h g^−1^ remaining after 300 cycles, is illustrated in Figure 6c. Remarkably, as shown in Figure 6d, the HMoO_x_−Zn||γ−MnO_2_ full cell maintained a high discharge capacity of 131 mAh g^−1^ after 300 cycles at 0.1 A g^−1^. Therefore, the HMoO_x_ coating could promote minimal electrode polarization and enhance cycling stability. Compared to the Zn anodes fabricated for preventing dendrite growth in the Zn anode (as reported in Table S1), the HMoO_x_−Zn anode remained at a relatively high level.

**Figure 7:**
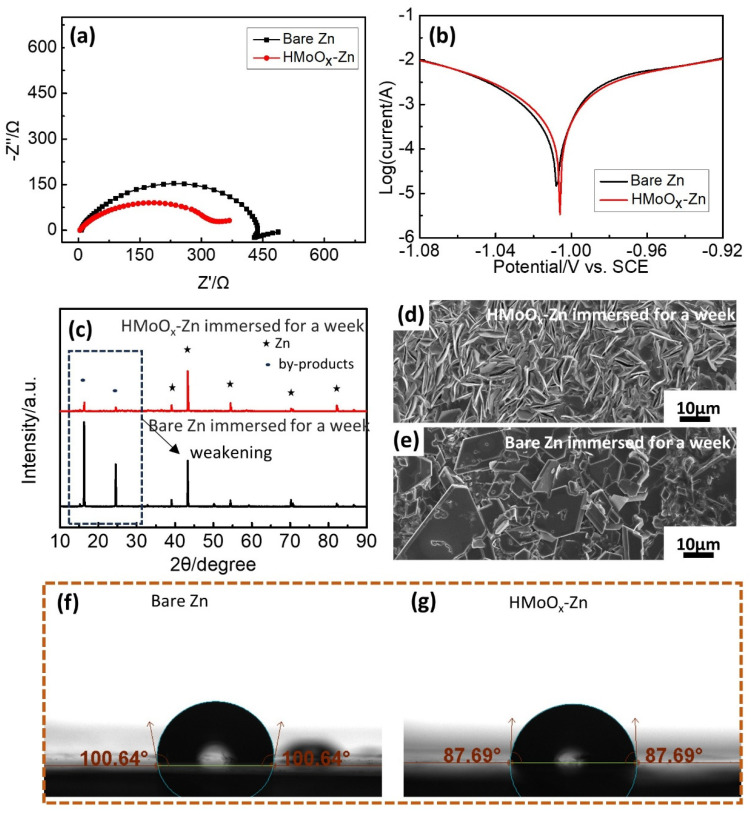
The analysis of property for the HMoO_x_−Zn anode. (**a**) The EIS spectra of bare Zn and HMoO_x_−Zn symmetrical batteries. (**b**) Polarization curves of bare Zn and HMoO_x_−Zn. (**c**) The XRD patterns and SEM of bare Zn. (**d**,**e**) The SEM of HMoO_x_−Zn foils after immersion in the ZnSO_4_ aqueous electrolyte for one week. (**f**,**g**) The contact angles of the electrolyte on bare Zn and HMoO_x_−Zn.

EIS plots were measured to assess the Zn^2+^ plating kinetics in the symmetric cells. Figure 7a depicts the EIS data of the bare Zn and HMoO_x_−Zn before cycle testing. Evidently, the HMoO_x_−Zn symmetric cells demonstrated smaller charge transfer resistance (Rct) in comparison to the bare Zn symmetric cells, suggesting that applying a HMoO_x_ layer on the zinc could enhance the migration charge of ions, potentially alleviating the degradation of cycle performance due to polarization. This was further confirmed by the analysis results obtained from the polarization curves shown in Figure 7b. The overpotential of HMoO_x_−Zn was higher than that of the bare Zn, and its corrosion current was lower than that of the bare Zn, suggesting that the presence of the HMoO_x_ layer could function as a corrosion inhibitor, guarding the zinc anode against corrosion [50]. In order to assess the corrosion resistance of zinc metal following surface coating modification, bare zinc foils and HMoO_x_−Zn plates were submerged in a 1 M ZnSO_4_ aqueous electrolyte for one week. As illustrated in Figure 7c, for the Zn anode and HMoO_x_−Zn anode, similar diffraction peaks of new phase by−products at 16.2° and 24.6° were detected after a soaking state for a week, but the relative peak intensities of the HMoO_x_−Zn anode were weaker than those of the bare Zn anode. This indicated that the HMoO_x_ layer might act as a safeguard, diminishing the rate of corrosion for zinc in the aqueous electrolyte. The SEM image in Figure 7e displayed a high number of large and irregular flaky products loosely gathered on the exposed zinc surface, indicating a significant level of chemical corrosion occurring on the surface of the zinc metal in the aqueous electrolyte. Additionally, these corrosion products could significantly hinder electron/ion diffusion, elevate interface impedance, and consequently obstruct zinc plating/stripping reactions, whereas when coated with HMoO_x_, only a few flakes were observed, and they were significantly smaller in size (as depicted in Figure 7d), indicating that the zinc corrosion was effectively suppressed. To investigate the impact of a HMoO_x_ coating on Zn plates, contact angles were measured (see Figure 7f,g). The contact angles of HMoO_x_−Zn and bare zinc were measured at 87.69° and 100.64°, respectively. The lower contact angle of HMoO_x_−Zn demonstrated its superior surface wetting properties. Improving surface wettability enhanced the effective interaction between the electrolyte and the HMoO_x_−Zn anode while also aiding in reducing charge transfer resistance. This, in turn, promoted the migration of Zn^2+^ ions and ensured uniform deposition [41,51]. Consequently, the prevention of zinc dendrite formation was achieved.

**Figure 8:**
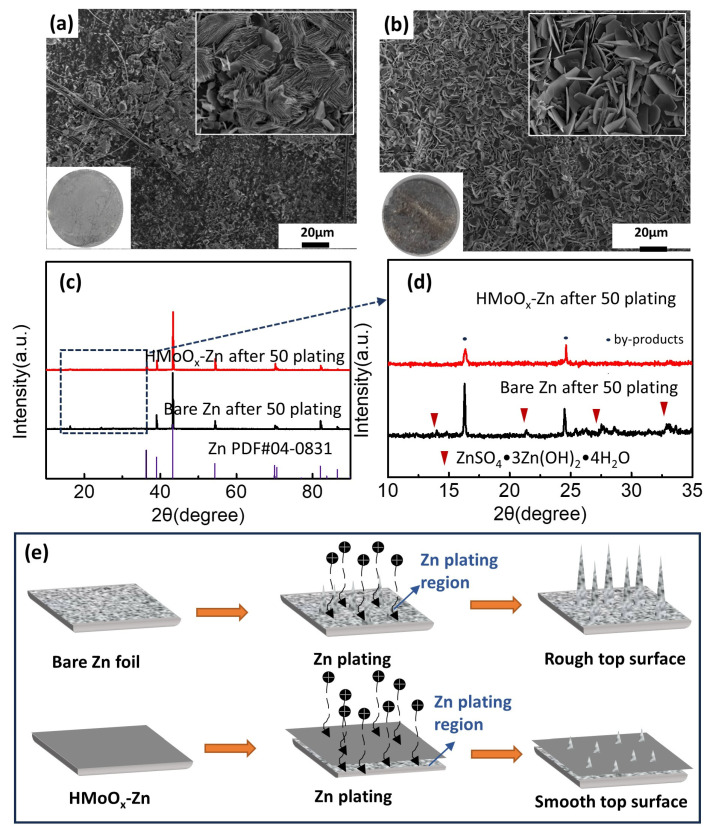
Mechanism of performance improvement for the HMoO_x_−Zn anode. (**a**,**b**) The SEM images of the bare Zn and HMoO_x_−Zn after 50 cycles. The insert graphs are digital photographs corresponding to Figure 8a,b. (**c**) The XRD patterns for bare Zn and HMoO_x_−Zn after 50 plating cycles. (**d**) The high−resolution XRD patterns in Figure 8c are shown at low angles. (**e**) The schematic diagram of the dendrite inhibition mechanism of the HMoO_x_ coating.

The characterizations of bare Zn and HMoO_x_−Zn in the plating/stripping process are examined in Figure 8. SEM imaging was utilized to investigate the morphological changes in both electrodes following various states of stripping and plating, as presented in Figure 8a,b. After 50 cycles, it was obvious that the bare Zn plate exhibited an abundance of dendrites that may have developed and partially breached the separator, while the homogeneous deposition layer on the surface of HMoO_x_−Zn appeared to consist primarily of densely packed flake−like products. We speculated that the dense phase observed atop the HMoO_x_ layer comprised deposited zinc metal, and the irregular flakes were determined as sulfate by−products. The XRD patterns of the bare Zn and HMoO_x_−Zn after 50 cycles are displayed in Figure 8c. It can be observed that the crystalline structures of both electrodes had no notable discrepancies, indicating that zinc plating and stripping were the predominant electrochemical reactions detected throughout the cycling process. However, several newly emerged diffraction peaks of the two electrodes corresponded to ZnSO_4_·3 Zn(OH)_2_·4 H_2_O in Figure 8d, illustrating that side reactions, such as corrosion and hydrogen evolution, took place in both anodes during cycling [52]. Moreover, the protective layer can promote uniform Zn deposition, and this reduction in dendrite growth consequently decreased the by−products associated with dendrite formation; therefore, the peaks of Zn by−products in the modified Zn anode were lower than those in the bare Zn. Based on the preceding experimental characterization, it could be inferred that the presence of the HMoO_x_ layer substantially impeded dendrite growth and reduced the incidence of side reactions. In addition to its wettability, the total performance was improved. However, zinc dendrites grew vigorously on bare zinc foil without any protective measures because of the impact of the tip effect, as displayed in Figure 8e. Hence, the presence of the HMoO_x_ layer aided in safeguarding the zinc anode by inducing uniform deposition of Zn^2+^ and facilitating Zn^2+^ migration.

**Figure 9:**
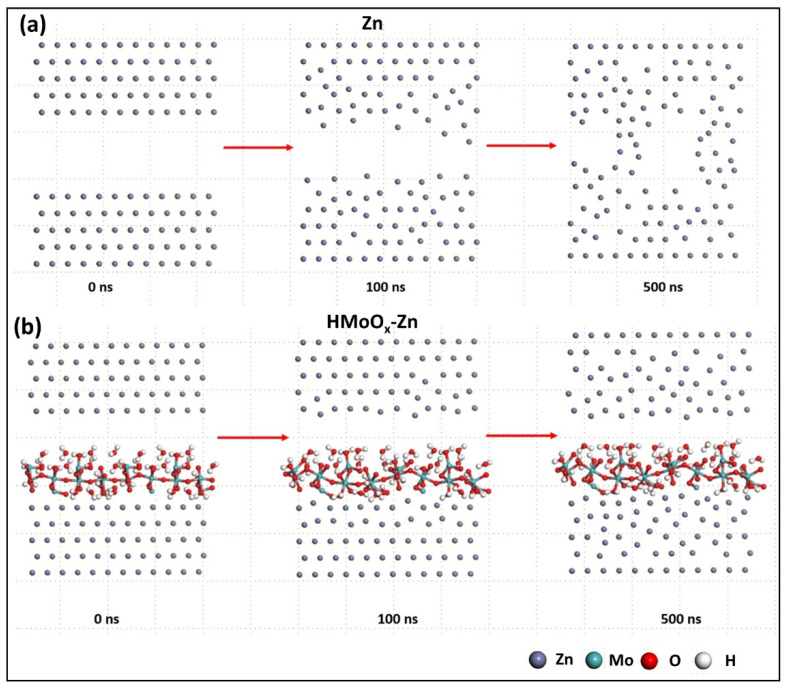
Theoretical calculation verification of the performance improvement for the HMoO_x_−Zn anode. The molecular dynamics simulations for the surface of the Zn anode (**a**) and HMoO_x_−Zn anode (**b**).

In order to further illustrate the importance of the HMoO_x_ coating, we performed two sets of molecular dynamics simulations for the surfaces of the HMoO_x_−Zn and Zn anodes. Figure 9a,b show the surface structures of both the Zn and HMoO_x_−Zn anodes in the range of 0–500 ns, respectively. The presence of HMoO_x_ suppressed the growth of Zn dendrites by forming a protective layer by interacting with Zn ions and preventing unstable reactions in localized regions. This inhibition potentially helped maintain surface uniformity and reduced dendrite formation. Additionally, HMoO_x_ may have formed a barrier layer on the surface of the Zn anode, impeding the free migration of Zn ions. This barrier layer effectively slowed down the rate of dendrite expansion. The results of these simulations were consistent with the above experimental outcomes, which fully demonstrated the protective role of the HMoO_x_ layer on the performance of the Zn anode.

## 3. Materials and Methods

### 3.1. Materials’ Synthesis

For synthesizing zinc foils coated with HMoO_x_, the Zn foils were purchased from Hengxing Trade Co., Ltd. (Cardiff, UK) before conducting the experiments. The surface of the bare Zn was cleaned using diluted hydrochloric acid and ethanol, respectively. The HMoO_x_ was electrochemically deposited onto the Zn sheet using a simple three−electrode system. The working electrode employed was a zinc sheet measuring 30 μm in thickness and cut to dimensions of 2.0 × 2.5 cm^2^, while the deposition area was 4 cm^2^ to ensure the same deposition area. The reference electrode utilized was the SCE electrode, with platinum foil functioning as the counter electrode. A 5 mM solution of ammonium molybdate (Sino Pharm, Beijing, China) was utilized as the electrolyte. The space between the zinc sheet and the Pt electrode was approximately 1.5 cm. The HMoO_x_ layer was electrodeposited onto the zinc foil by maintaining a potential of −0.8 V for durations of 0, 20, 50, 75, 100, 150, 200, and 250 s. The Zn coated with HMoO_x_ underwent multiple rinses with deionized water/ethanol and was subsequently subjected to vacuum drying at 60 °C.

### 3.2. Materials’ Characterizations

X−ray diffraction (XRD, Brucker, D8 Advances, Karlsruhe, Germany) was employed to study the crystallographic phase of the samples. Scanning electron microscopy (SEM; Regulus 8100, Hitachi, Japan) was carried out to study the morphology of the samples. The contact angles of electrolyte droplets (1 M ZnSO_4_) on the bare zinc and HMoO_x_−coated zinc were measured using the Dataphysics DCAT21 instrument. X−ray photoelectron spectroscopy (XPS; Shimadzu AXIS ULTRA DLD, Kyoto, Japan) was utilized to determine the chemical or electronic configurations of elements

### 3.3. Electrochemical Measurements

To evaluate the plating/stripping behaviors and cyclic durability of the bare Zn and HMoO_x_−Zn anodes in charge and discharge processes, symmetric cell setups were created by pairing identical HMoO_x_−Zn anodes (or bare Zn foils) measuring 16 mm in diameter. The glass fiber was employed as a separator (Whatman, Grade GF/A, English) and ZnSO_4_ aqueous solution (1 M) was used as the electrolyte. The long−term cycle and rate performance of symmetrical batteries were tested under different current densities utilizing the NEWARE battery testing instrument. The corrosion behavior was assessed via linear polarization, employing HMoO_x_−Zn (or bare Zn) for both the counter and working electrodes, and utilizing SCE as the reference electrode in a 1 M ZnSO_4_ solution, with a scan rate set at 5 mV s^−1^.

To assess the electrochemical performance of HMoO_x_−Zn/γ−MnO_2_ (bare Zn/γ−MnO_2_) full batteries, a slurry was prepared by mixing the active materials γ−MnO_2_ (Chizhou Jingyan New Energy Technology Co., Ltd., Chizhou, Anhui province, China > 99.0%), acetylene black, and polyvinyl difluoride in a weight ratio of 8:1:1, along with a suitable quantity of N−methyl pyrrolidone solvent. The mixture was subsequently spread onto a titanium foil and dried under vacuum conditions at 90 °C for a period of 18 h. Circular electrodes with a diameter of 12 mm were cut from the sheets, achieving a calculated mass loading of approximately 0.93 mg cm^−2^ for γ−MnO_2_. The full cells were assembled into CR2025 coin cells under ambient atmospheric conditions, featuring HMoO_x_−Zn (or bare Zn) foil anodes and γ−MnO_2_ cathodes, separated by a 19 mm−diameter glass fiber separator, and utilizing a 1 M ZnSO_4_ aqueous solution as the electrolyte. Galvanostatic charge/discharge processes were performed by the NEWARE battery testing instrument within the voltage range of 1.0–1.8 V (vs. Zn^2+^/Zn). The electrochemical workstation (CHI660E, Shanghai Chenhua Instrument Co., Ltd, Shanghai, China) was utilized for conducting electrochemical impedance spectroscopy (EIS) and cyclic voltammetry (CV). All test conditions were controlled at 25 °C unless otherwise specified.

### 3.4. Theoretical Calculations

The VASP code was used to perform density functional theory (DFT) calculations [53]. Under generalized gradient approximation (GGA), the Perdew–Burke–Ernzerhof (PBE) functional was employed to compute the exchange correlation [54]. In order to describe the expansion of the electronic eigenfunctions, a projector−augmented wave (PAW) pseudopotential, configured with a kinetic energy cut−off of 500 eV, was utilized [55]. The vacuum thickness was adjusted to 15 Å in order to minimize interlayer interactions. The Γ−centered 5 × 5 × 1 Monkhorst–Pack k points were utilized for sampling the Brillouin−zone integration, and all atomic positions underwent complete relaxation until energy and force, respectively, reached a tolerance of 1 × 10^−5^ eV and 0.03 eV/Å. The long−range interactions were illustrated by the dispersion−corrected DFT−D method [56].

## 4. Conclusions

In summary, a HMoO_x_ coating was synthesized on the zinc surface via a straightforward electrodeposition method, employed as the anode material for aqueous ZIBs. The HMoO_x_ coating effectively mitigated corrosion and suppressed side reactions during Zn deposition/stripping. It also improved surface wettability and reduced the interfacial impedance, which was beneficial to facilitate Zn^2+^ migration and uniform deposition of Zn^2+^, thereby inhibiting dendrite formation on the zinc anode. The symmetric cell with the HMoO_x_−coated zinc anode demonstrated remarkable long−term cycle stability, maintaining an exceptionally low−voltage hysteresis of 80 mV at 2.5 mA cm^−2^ over 3000 cycles. Additionally, the HMoO_x_−Zn||γ−MnO_2_ full cell exhibited a notably enhanced cycle and rate performance, achieving a high discharge capacity of 131 mAh g^−1^ after 300 cycles at 0.1 A g^−1^. Moreover, the simulation of the HMoO_x_−Zn anode verified the protective role of the HMoO_x_ layer on the performance of the zinc anode. This research offers an innovative approach for designing dendrite−free zinc anodes in high−performance, metal−based batteries and can also be extended to the protection of other metal anodes.

## Data Availability

Data are contained within the article and Supplementary Materials.

## Supplementary Materials

The following supporting information can be downloaded at: https://www.mdpi.com/article/10.3390/molecules29133229/s1, Figure S1: Galvanostatic cycling performances of bare Zn symmetrical cells with at different deposition times of 20 s, 50 s, 75s, 100 s, 150 s, 200 s at a current density of 2.5 mA cm−2; Figure S2: (a,b) The SEM of HMoOx-Zn anode at different deposition times of 20 s and 100 s; Figure S3: The TEM of HMoOx at various magnification. Table S1: Comparison reported Zn anodes fabricated for preventing dendrite growth.
