# Supplementary material for: Hydrous Molybdenum Oxide Coating of Zinc Metal Anode via the Facile Electrodeposition Strategy and Its Performance Improvement Mechanisms for Aqueous Zinc−Ion Batteries
Source: Molecules. 2024 Jul 8;29(13):3229. doi: 10.3390/molecules29133229 (PMC11243360; doi:10.3390/molecules29133229)
Supplement: Supplementary file 1 [file molecules-29-03229-s001.zip › molecules-3073861-supplementary.pdf]

Hydrous Molybdenum Oxide Coating of Zinc Metal Anode via the Facile Electrodeposition Strategy and Its Performance Improvement Mechanisms for Aqueous Zinc−Ion Batteries

Jianwei Yuan, Yutao Shi, Weibai Bian, Huaren Wu, Yingjun Chen, Chengcheng Zhou, Xiaohui Chen *, Wei Zhang and Hailin Shen *

School of Chemical Engineering and Materials, Changzhou Institute of Technology, Changzhou 213022, China; yuanjw@czust.edu.cn (J.Y.)

**Correspondence: chenxh@czust.edu.cn (X.C.); shenhailin@czust.edu.cn (H.S.)*

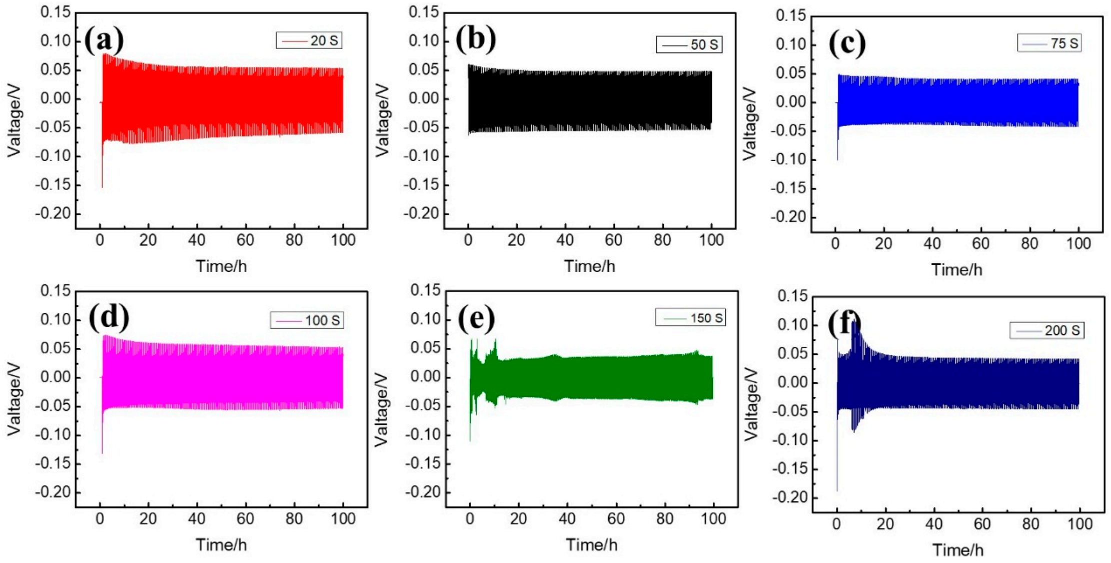

Figure S1. Galvanostatic cycling performances of bare Zn symmetrical cells with at different deposition times of 20 s, 50 s, 75s, 100 s, 150 s, 200 s at a current density of 2.5 mA cm^{-2}.

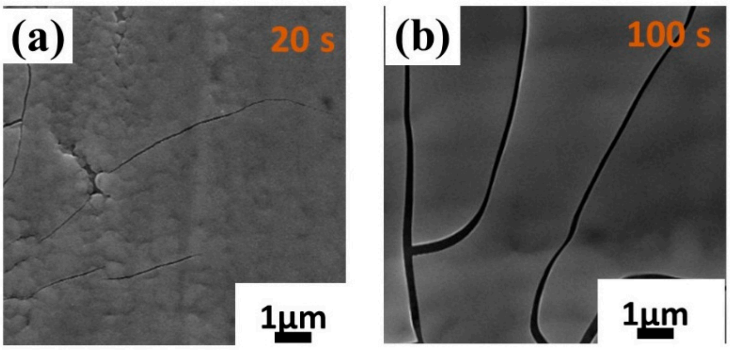

Figure S2. (a, b) The SEM of $HMoO_x$-Zn anode at different deposition times of 20 s and 100 s.

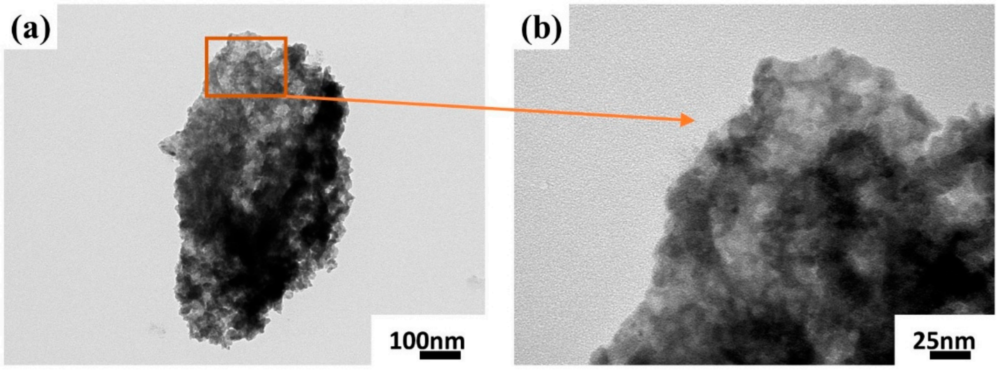

Figure S3. The TEM of $HMoO_x$ at various magnification.

Table S1. Comparison reported Zn anodes fabricated for preventing dendrite growth in Zn anode.

Anode	Current density (A/g)	Final retention capacity [mAh/g]	Number of cycles	Ref.
Zn@$CaCO_3$//MnO_2	1	177	1000	[1]
Zn@ZnO-3D//MnO_2	0.5	212.9	500	[2]
3D Zn@carbon// MnO_2	0.308	140	200	[3]
TiO_2@Zn//MnO_2	0.5	100	300	[4]
CeO_2@Zn//MoS_2	2	90	300	[5]
Zn@graphene//Li$x$$MnO_2$	1	90	300	[6]
Zn//γ-MnO_2	0.1	50	300	This work
$HMoO_x$-Zn//γ-MnO_2	0.1	131	300	This work
